# Ferrostatin-1 alleviates lipopolysaccharide-induced acute lung injury via inhibiting ferroptosis

**DOI:** 10.1186/s11658-020-00205-0

**Published:** 2020-02-27

**Authors:** Pengfei Liu, Yetong Feng, Hanwei Li, Xin Chen, Guangsuo Wang, Shiyuan Xu, Yalan Li, Lei Zhao

**Affiliations:** 1 Department of Anesthesiology, The 2nd Clinical Medical College (Shenzhen People’s Hospital) of Jinan University, The 1st Affiliated Hospitals of Southern University of Science and Technology, Shenzhen, 518020 China; 2 Integrated Chinese and Western Medicine Postdoctoral Research Station, Jinan University, Guangzhou, 510632 China; 3 Health Science Center, School of Basic Medical Sciences, Shenzhen University, Shenzhen, 518037 China; 4 Department of Anesthesiology, Zhujiang Hospital of Southern Medical University, Guangzhou, 510280 China; 5 Department of Laboratory Medicine, The 2nd Clinical Medicine College (Shenzhen People’s Hospital) of Jinan University, The 1st Affiliated Hospitals of Southern University of Science and Technology, Shenzhen, 518020 China; 6 Department of Thoracic Surgery, The 2nd Clinical Medicine College (Shenzhen People’s Hospital) of Jinan University, The 1st Affiliated Hospitals of Southern University of Science and Technology, Shenzhen, 518020 China; 7 Department of Anesthesiology, First Affiliated Hospital of Jinan University, Guangzhou, 510632 China

**Keywords:** Ferrostatin-1, Ferroptosis, Lipopolysaccharide, Acute lung injury

## Abstract

**Background:**

Ferroptosis is a newly recognized type of cell death, which is different from traditional necrosis, apoptosis or autophagic cell death. However, the position of ferroptosis in lipopolysaccharide (LPS)-induced acute lung injury (ALI) has not been explored intensively so far. In this study, we mainly analyzed the relationship between ferroptosis and LPS-induced ALI.

**Methods:**

In this study, a human bronchial epithelial cell line, BEAS-2B, was treated with LPS and ferrostatin-1 (Fer-1, ferroptosis inhibitor). The cell viability was measured using CCK-8. Additionally, the levels of malondialdehyde (MDA), 4-hydroxynonenal (4-HNE), and iron, as well as the protein level of SLC7A11 and GPX4, were measured in different groups. To further confirm the in vitro results, an ALI model was induced by LPS in mice, and the therapeutic action of Fer-1 and ferroptosis level in lung tissues were evaluated.

**Results:**

The cell viability of BEAS-2B was down-regulated by LPS treatment, together with the ferroptosis markers SLC7A11 and GPX4, while the levels of MDA, 4-HNE and total iron were increased by LPS treatment in a dose-dependent manner, which could be rescued by Fer-1. The results of the in vivo experiment also indicated that Fer-1 exerted therapeutic action against LPS-induced ALI, and down-regulated the ferroptosis level in lung tissues.

**Conclusions:**

Our study indicated that ferroptosis has an important role in the progression of LPS-induced ALI, and ferroptosis may become a novel target in the treatment of ALI patients.

## Background

Acute lung injury (ALI) is regarded as a kind of critical clinical syndrome. It is also a disorder of acute inflammation, which causes interstitial edema, the accumulation of neutrophils as well as injury of the alveolar epithelium in the lung tissues [1–3]. Numerous studies have indicated that gram-negative bacterial infections are among the most important causes of ALI, and lipopolysaccharide (LPS) can lead to the lung injury and inflammatory response, which acts as the major component of outer membranes of gram-negative bacteria [4–7]. In recent years, LPS has been most widely used in the field of drug-associated ALI models, which can effectively induce a neutrophilic inflammatory response with an increase in intrapulmonary cytokines. In addition, LPS is considered as a potent activator of the innate immune responses via TLR4 pathways. Thus, the use of LPS provides information about the effects of host inflammatory responses, which occur in bacterial infections [8, 9]. Researchers have demonstrated that the intratracheal administration of LPS can induce the production of inflammatory mediators and reactive oxygen species (ROS), and worsen the lung tissue injury in an experimental animal model of ALI [10–13]. Therefore, the development of a novel treatment mode against LPS-induced ALI, which is based on inhibition of inflammation and oxidative stress, has attracted scientists’ attention in both clinical and pre-clinical studies.

Different from apoptosis, necrosis or autophagic cell death, ferroptosis is considered as a novel type of cell death, which mainly results from iron-dependent lipid peroxidation, and is characterized by mitochondrial shrinkage. Emerging evidence suggests that ferroptosis can be induced by down-regulation of system Xc^−^ activity, inhibition of glutathione peroxidase 4 (GPX4), and an increase of lipid ROS [14–17]. Many diseases have been demonstrated to be associated with ferroptosis, such as Alzheimer’s disease [18], carcinogenesis [19, 20], intracerebral hemorrhage [21], traumatic brain injury [22], stroke [23], and ischemia-reperfusion injury [24]. In addition, the relationship between ferroptosis and lung injury or other lung diseases has been investigated by some groups recently. In 2019, Li et al. found that ferroptosis holds a key role in radiation-induced lung fibrosis. Their results indicated that liproxstatin-1, a ferroptosis inhibitor, could alleviate radiation-induced lung fibrosis via down-regulation of TGF-β1 and activation of the Nrf2 signaling pathway, providing a novel therapeutic target for patients with radiation-induced lung fibrosis. Moreover, they also investigated the position of ferroptosis in the process of acute radiation-induced lung injury. Their study showed that obvious ferroptotic characteristic changes of mitochondria were observed in the acute radiation-induced lung injury model, and the level of glutathione peroxidase 4, a key marker of ferroptosis, was also decreased in this model, and it could be significantly alleviated by a ferroptosis inhibitor [25, 26]. Therefore, ferroptosis also played a crucial role in the acute radiation-induced lung injury. However, the detailed position of ferroptosis is still unclear for us in LPS-induced ALI.

In the present study, we mainly analyzed the role of ferroptosis in LPS-induced ALI in vitro and in vivo. We found that ferroptosis could play a critical role in LPS-induced ALI, and the ferroptosis inhibitor ferrostatin-1 (Fer-1) effectively alleviated LPS-induced ALI. Therefore, our study provided more insights into the cell death pathways in LPS-induced ALI and established a novel therapeutic approach for patients with ALI.

## Methods

### Cell culture

Cells from the human bronchial epithelial cell line BEAS-2B (ATCC, USA) were cultured with BEGM Bronchial Epithelial Cell Growth Medium BulletKit (Lonza) in a humidified incubator at 37 °C with 5% CO_2_. In addition, the culture medium was changed every other day. BEAS-2B cells were passaged (dilution, 1:3) every 3 or 4 days. In addition, air-liquid interface culture of BEAS-2B cells was performed as the reference [27].

### Cell viability assay

To evaluate cell viability, the CCK-8 (Dojindo) method was used in our study as the references [28, 29]. In brief, BEAS-2B cells were seeded into a 96-well plate at the concentration of 5 × 10^4^ cells/well. The cells were cultured for 24 h, then treated with LPS (Sigma) and Fer-1 (Sigma) in different concentrations for 16 h followed by the addition of 20 μl of CCK-8 solution directly into the medium (200 μl per well) and incubation at 37 °C for 4 h. The absorbances (Abs) in different groups were detected at 450 nm (*n* = 3). In the blank group, the well only contained medium, and the cells without any treatment were used as the control group. Herein, the cell viability = (Abs of experimental group-Abs of blank group)/(Abs of control group-Abs of blank group) × 100%.

### Western blot

In our study, the cell samples were lysed using radioimmunoprecipitation assay lysis buffer (RIPA, Thermo Fisher Scientific), and the total protein concentration of different groups was detected using the Pierce BCA Protein Assay Kit (Thermo Fisher Scientific). In our study, the cell lysates (20 μg/lane) were separated using 10% SDS-PAGE gel and then transferred to nitrocellulose membranes. The membrane was blocked with 5% nonfat dried milk diluted in PBS, and further incubated with primary antibodies overnight at 4 °C. Herein, the different primary antibodies used were: anti-SLC7A11 (1:3000; Cell signaling, Cat #: 12691), anti-GPX4 (1:1000; Santa Crus, Cat #: sc-166,570), anti-FTH (1:2000; Abcam, Cat #: ab65080) and anti-GAPDH (1:3000; Santa Cruz, Cat #: sc-47,724). The secondary antibodies used were: Anti-mouse IgG (HRP-conjugated; 1:5000; Sigma-Aldrich, Cat #: A-9044) and anti-rabbit IgG (HRP-conjugated; 1:5000; Sigma-Aldrich, Cat #: A-0545). Finally, the protein bands in each lane were visualized using SuperSignal West Femto Maximum Sensitivity Substrate (Thermo Fisher Scientific) and ChemiDoc Imagers (Bio-Rad Laboratories). The results were finally quantified using the ImageJ 1.x software (National Institutes of Health). All of the raw, uncropped blots for images throughout the paper are shown in Supplementary Fig. 1.

### Evaluation of malondialdehyde (MDA), 4-hydroxynonenal (4-HNE) and iron level

In our study, to evaluate the ferroptosis level in different groups, the MDA, 4-HNE and iron levels were detected in each group. The MDA concentration, 4-HNE concentration and iron concentration in cell lysates were assessed using the Lipid Peroxidation (MDA) Assay Kit (Sigma-Aldrich, Cat #: MAK085), Lipid Peroxidation (4-HNE) Assay Kit (Abcam, Cat #: ab238538) and Iron Assay Kit (Sigma-Aldrich, Cat #: MAK025) according to the manufacturer’s instructions.

### Real-time quantitative PCR (qRT-PCR)

The total RNA was extracted using TRIzol solution (Thermo Fisher Scientific). The cDNA of different samples was synthesized using 2 μg of total RNA as well as the Transcriptor first-strand cDNA synthesis kit (Promega). Then the qRT-PCR was performed with SYBR Green Master Mix (TAKARA). The sequences of different primers are as follows (5′ to 3′):

Mouse *Hepcidin* -F 5CTGCGCCTTTTCAAGGATGG.

Mouse *Hepcidin*-R AATTGTTACAGCATTTACAGCAGAAGA.

Mouse *Ptgs2*-F CTGCGCCTTTTCAAGGATGG.

Mouse *Ptgs2*-R GGGGATACACCTCTCCACCA.

Mouse *Actb*-F AAATCGTGCGTGACATCAAAGA.

Mouse *Actb*-R GCCATCTCCTGCTCGAAGTC.

Human *HEPCIDIN*-F CTGACCAGTGGCTCTGTTTTC.

Human *HEPCIDIN*-R GAAGTGGGTGTCTCGCCTC.

Human *ACTB*-F CCCAGAGCAAGAGAGG.

Human *ACTB*-R GTCCAGACGCAGGATG.

### Animal experiments

In our study, the male C57BL/6 mice were divided randomly into 4 groups (*n* = 4 per group, 8–10 weeks old, weight = 23–25 g): the control group receiving 0.9% NaCl (containing 0.1% DMSO), the LPS group receiving LPS plus 0.9% NaCl (containing 0.1% DMSO), the Fer-1 group receiving Fer-1 only, and the LPS + Fer-1 group receiving both Fer-1 and LPS. The LPS-induced ALI model was induced by instilling intratracheally 50 μl of LPS solution (0.2 g/L), then Fer-1 (0.8 mg/kg) was administered after LPS challenge via tail vein injection. The Fer-1 was dissolved in DMSO first, and diluted with 0.9% NaCl. The final concentration of Fer-1 and DMSO was 0.2 mg/ml and 0.1% respectively. After the treatments for 16 h, the mice in each group were euthanized and bronchoalveolar lavage (BAL) fluid was collected via lung lavage. To analyze the differential BAL cell counts, the cells were concentrated using a Cytospin 4 (Thermo Fisher Scientific). Cell staining was performed using the Shandon Kwik-Diff kit (Thermo Fisher Scientific). Additionally, the total protein concentration and the levels of IL-6 and TNF-α in each sample were detected with the Pierce BCA Protein Assay Kit (Thermo Fisher Scientific), IL-6 ELISA Kit ELISA kit (Invitrogen) and TNF-α ELISA Kit (Invitrogen) according to the manufacturer’s instructions. Lung tissues in different groups were collected for qPCR and western blot detection, and part of lung tissues was fixed using 10% buffered formalin, then the tissues were embedded in paraffin for histological analyses as the references [25, 30–32]. Herein, a scoring system of 0–4 was used for the evaluation of lung injury as the reference [33].

### Statistical analysis

In this study, all of the results are presented as mean ± SD. SPSS 17.0 software was used for statistical analysis. Herein, the difference between two groups was analyzed with unpaired Student’s t-test, and the difference among three or more groups was analyzed with one-way ANOVA with Bonferroni’s correction. A one-tailed test was used in Student’s t-test. *p* < 0.05 was considered statistically significant.

## Results

### LPS treatment promotes ferroptosis in BEAS-2B cells

**Fig. 1:**
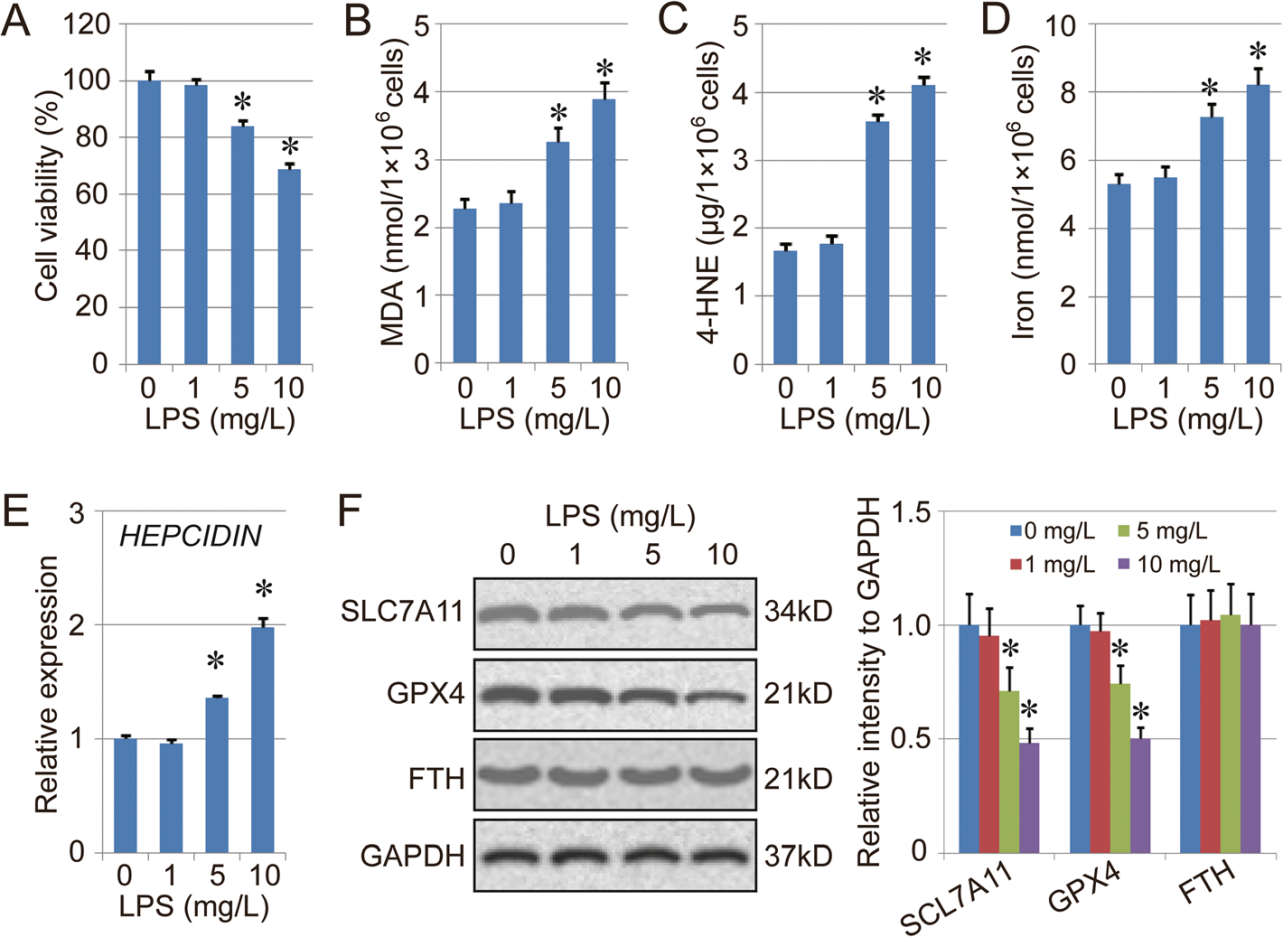
The effect of LPS treatment on ferroptosis in BEAS-2B cells. **a**. Cell viability of BEAS-2B cells treated with LPS. The cells were treated with LPS in different concentrations (1, 5, and 10 mg/L) for 16 h, then the cell viability of each group was measured using CCK-8. **b**-**d**. Levels of MDA (B), 4-HNE (C) and total iron (D) in the BESA-2B cells treated with LPS. **e**. mRNA expression of *HEPCIDIN*. **f**. Protein levels of SLC7A11 and GPX4 in the BESA-2B cells treated with LPS. Results are expressed as means±SEM (*n* = 3). *: *p* < 0.05 compared with the 0 mg/L group

To evaluate the effect of LPS treatment on ferroptosis, BEAS-2B cells were treated with LPS in different concentrations (1, 5 and 10 mg/L) for 16 h. Cell viability was detected using the CCK-8 method. The results showed that LPS treatment could inhibit cell viability in a dose-dependent manner (Fig. 1A). Also, the amount of MDA, 4-HNE and total iron in the cells treated with LPS was increased significantly (Fig. 1b-d). Some reports have indicated that LPS induces iron overload in vivo and in vitro [34, 35], and the up-regulation of HEPCIDIN could be the key mechanism during this process. We detected the level of *HEPCIDIN* and ferritin heavy chain (FTH) in this study, and the results indicated that expression of *HEPCIDIN* was increased in BEAS2B cells treated with LPS. However, no significant difference in FTH expression was found between the control group and LPS treatment groups (Fig. 1e-f). Therefore, the iron overload should be the key reason for up-regulation of total iron. In addition, the protein levels of two ferroptosis markers, SLC7A11 and GPX4, were also evaluated by western blot. The results indicated that the expression of both SLC7A11 and GPX4 was down-regulated by LPS treatment, suggesting that LPS treatment promotes ferroptosis in BEAS-2B cells (Fig. 1f).

### Fer-1 attenuates LPS-induced cell injury via inhibiting ferroptosis

**Fig. 2:**
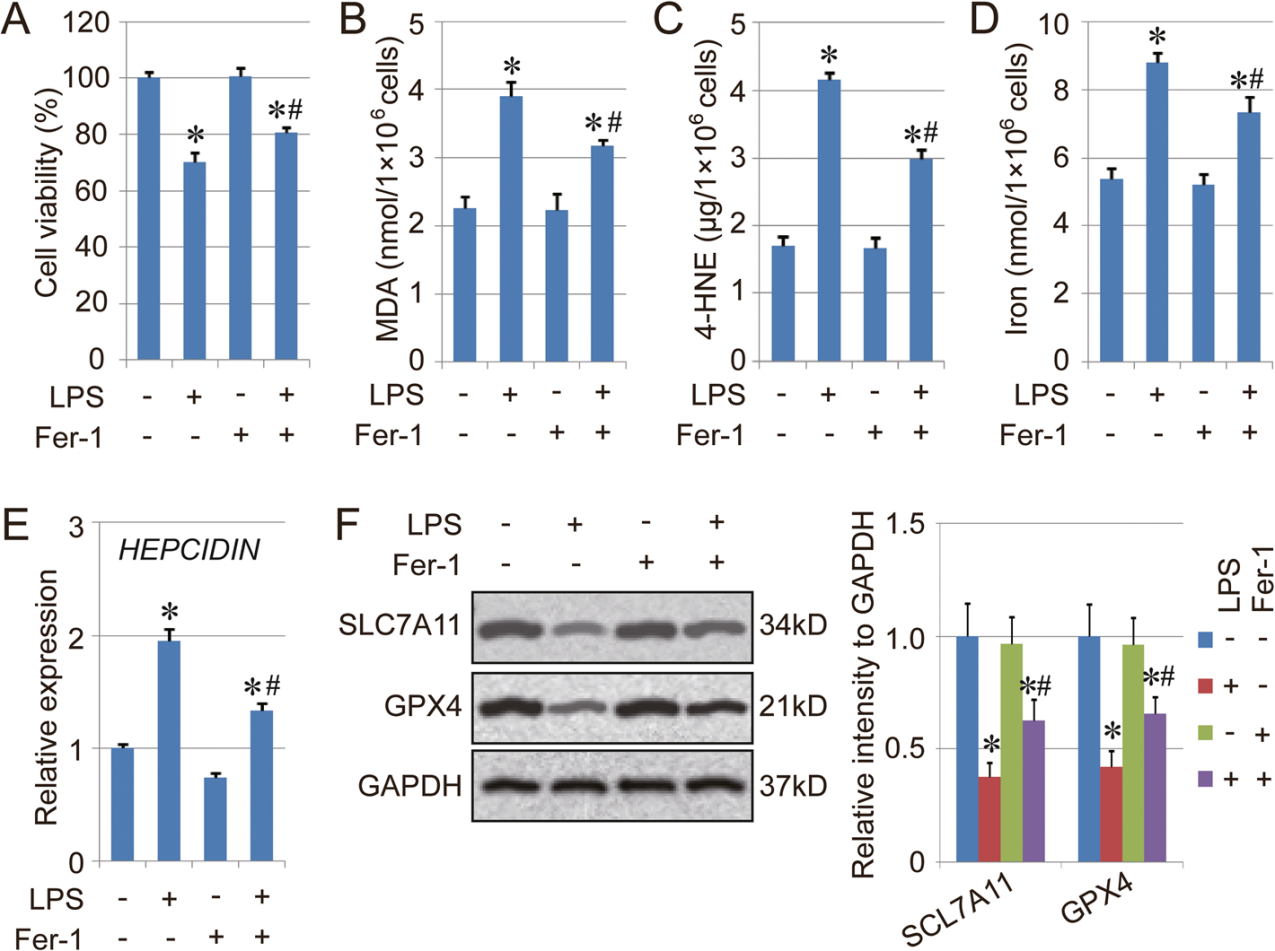
Fer-1 attenuates LPS-induced cell injury. **a**. Cell viability of BEAS-2B cells treated with LPS and Fer-1. The cells were treated with LPS (10 mg/L) and Fer-1 (2 μM) for 16 h, then the cell viability of each group was measured using CCK-8. **b**-**d**. Levels of MDA (B), 4-HNE (C) and total iron (D) in the BESA-2B cells treated with LPS. **e**. mRNA expression of *HEPCIDIN*. **f**. Protein levels of SLC7A11 and GPX4 in the BESA-2B cells treated with LPS. Results are expressed as means±SEM (*n* = 3). *: *p* < 0.05 compared with the control group. #: *p* < 0.05 compared with the LPS group

To further confirm the effect of LPS on ferroptosis regulation, Fer-1, a ferroptosis inhibitor, was applied in our study. We found that the co-treatment of LPS and Fer-1 still showed inhibition of cell viability. However, the cell viability in the LPS + Fer-1 group was higher than the LPS group, indicating the rescue effect of Fer-1 on LPS-induced cell death (Fig. 2a). In addition, the amounts of MDA, 4-HNE and total iron in the LPS + Fer-1 group were also lower than those in the LPS group (Fig. 2b-d). The mRNA level of *HEPCIDIN* in the LPS group also could be decreased by Fer-1 treatment in vitro (Fig. 2e). Moreover, the expression of both SLC7A11 and GPX4 was up-regulated in the LPS + Fer-1 group compared with the LPS group (Fig. 2f). However, the treatment with Fer-1 (Fer-1 group) did not affect cell viability or cell ferroptosis in normal BEAS-2B cells, which could be because of the low basal level of ferroptosis in normal cells. Overall, those results suggested the key role of ferroptosis in LPS-induced cell injury.

**Fig. 3:**
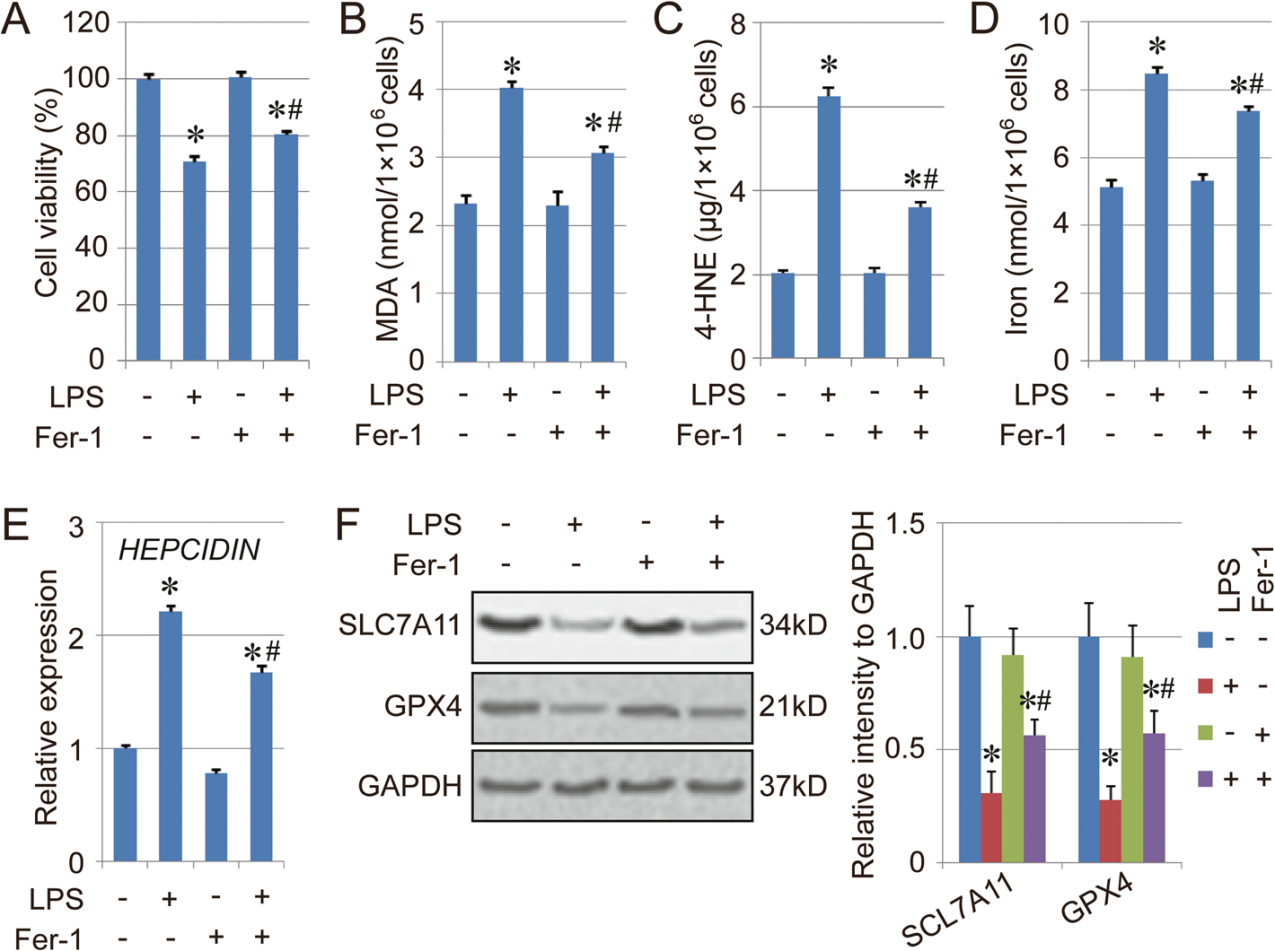
Effect of Fer-1 on LPS-induced cell injury in an air-liquid interface. **a**. Cell viability of BEAS-2B cells in an air-liquid interface treated with LPS and Fer-1. The cells were treated with LPS (10 mg/L) and Fer-1 (2 μM) for 16 h, then the cell viability of each group was measured using CCK-8. **b**-**d**. Levels of MDA (B), 4-HNE (C) and total iron (D) in the BESA-2B cells treated with LPS. **e**. mRNA expression of *HEPCIDIN*. **f**. Protein levels of SLC7A11 and GPX4 in the BESA-2B cells treated with LPS in an air-liquid interface. Results are expressed as means±SEM (*n* = 3). *: *p* < 0.05 compared with the control group. #: *p* < 0.05 compared with the LPS group

To simulate the half-way physiological behavior of airway epithelial cells, BEAS-2B cells grown in an air-liquid interface were used to confirm the role of ferroptosis in LPS-induced cell injury. Similar to BEAS-2B cells cultured in normal conditions, the viability of the cells grown in an air-liquid interface was decreased by LPS treatment, which could be relieved to some degree by Fer-1 (Fig. 3a). Moreover, the levels of MDA, 4-HNE and total iron, as well as the expression of *HEPCIDIN*, in the LPS + Fer-1 group were lower than in the LPS group (Fig. 3b-e), and the expression of both SLC7A11 and GPX4 was higher in the LPS + Fer-1 group than the LPS group (Fig. 3f), indicating the rescue effect of Fer-1 in LPS-induced cell injury.

### Therapeutic action of Fer-1 against LPS-induced ALI

**Fig. 4:**
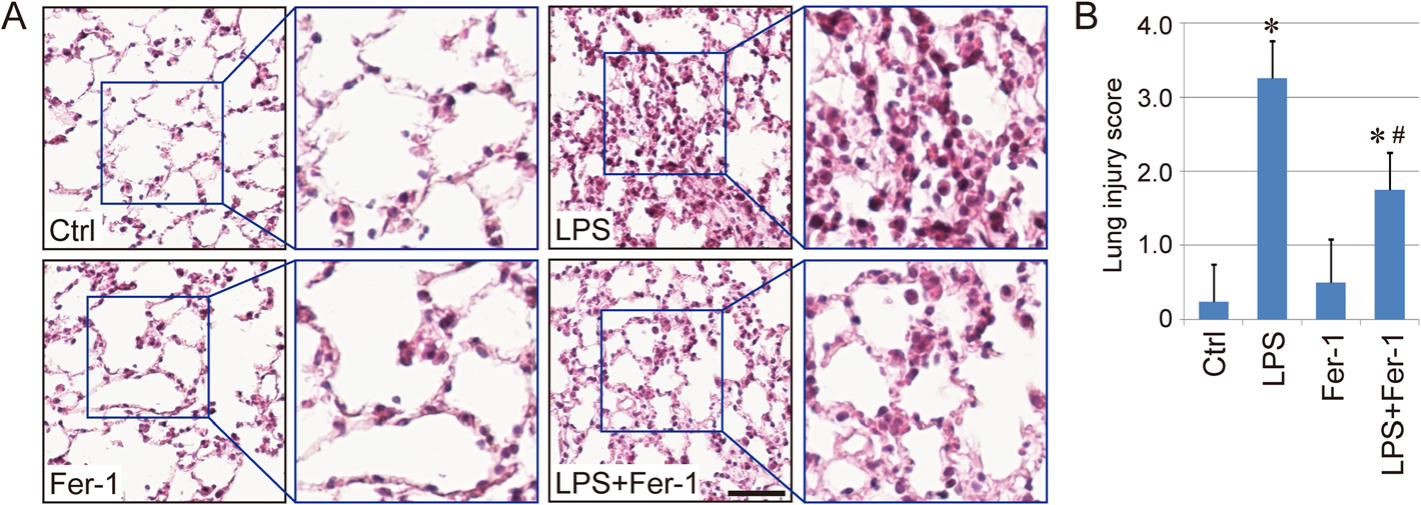
Therapeutic action of Fer-1 against LPS-induced ALI. **a**. Hematoxylin and eosin (HE) staining of lung tissue sections from different groups (Scale bar = 50 μm). The raw images of HE staining are shown in Supplementary Fig. 2. **b**. Lung injury score of mice in each group. Results are expressed as means±SEM (*n* = 4). *: *p* < 0.05 compared with the control group. #: *p* < 0.05 compared with the LPS group

**Fig. 5:**
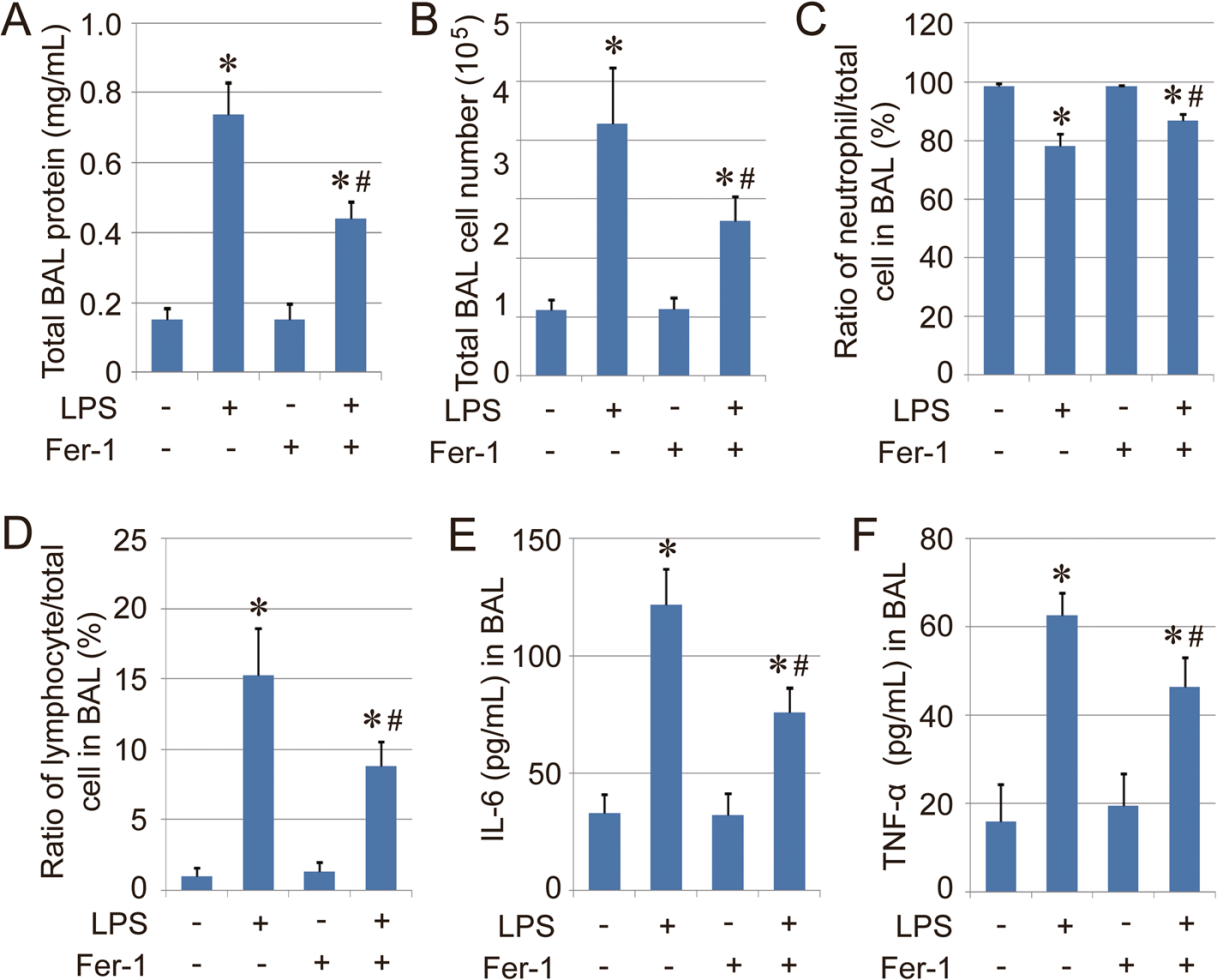
BAL assay. **a**. Total protein concentration in BAL fluid from each group. **b**. Total BAL cell numbers in each. **c**-**d**. Percentage of BAL neutrophils and BAL lymphocytes in different groups. **e**-**f**. Concentration of IL-6 and TNF-α in each group. Results are expressed as means±SEM (*n* = 4). *: *p* < 0.05 compared with the control group. #: *p* < 0.05 compared with the LPS group

The therapeutic action of Fer-1 against LPS-induced ALI was further evaluated in vivo using a mouse model. The mice were exposed to an LPS-induced model of inflammatory lung injury, and both BAL fluid and lung tissues were collected for evaluation. The results indicated that the mice in LPS groups exhibited the greatest degree of injury, followed by the LPS + Fer-1 group. No obvious injury was found in either the control group or the Fer-1 group (Fig. 4a-b). The levels of BAL protein and the number of BAL cells were measured, and the results also indicated the relief of the inflammatory response in the LPS + Fer-1 group compared with the LPS group (Fig. 5a-b), which was further confirmed by the differential BAL cell counts (Fig. 5c-d), as well as the levels of BAL proinflammatory cytokines IL-6 and TNF-α (Fig. 5e-f). Therefore, these results indicated that the ferroptosis inhibitor Fer-1 exerts therapeutic action against LPS-induced ALI.

### Fer-1 alleviates LPS-induced ALI via inhibiting ferroptosis

**Fig. 6:**
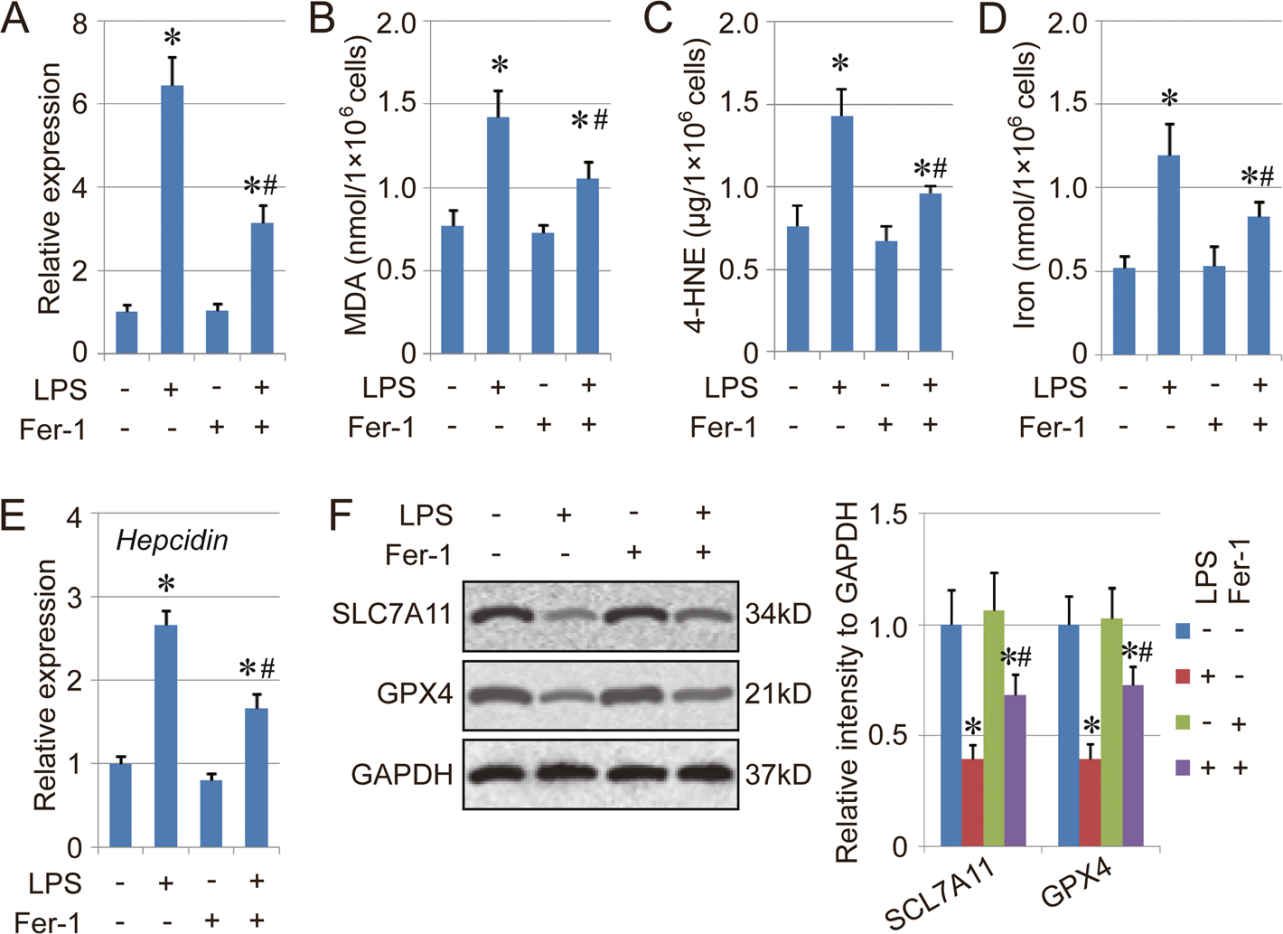
Fer-1 alleviates LPS-induced ALI through regulating ferroptosis. **a**. The qPCR analysis of the expression of Ptgs2 in each group. **b**-**d**. Levels of MDA (B), 4-HNE (C) and total iron (D) in the lung tissues of different groups. **e**. mRNA expression of *HEPCIDIN*. **f**. Protein levels of SLC7A11 and GPX4 in the lung tissues of different groups. Results are expressed as means±SEM (*n* = 4). *: *p* < 0.05 compared with the control group. #: *p* < 0.05 compared with the LPS group

The ferroptosis level in lung tissue was evaluated to analyze the effect of Fer-1. The qPCR results of mouse *Ptgs2* (prostaglandin endoperoxide synthase 2), which is a marker for the assessment of ferroptosis in vivo, suggested that LPS treatment promoted ferroptosis in lung tissues, which was alleviated partially by co-treatment with Fer-1 (Fig. 6a). Similarly, the levels of MDA, 4-HNE and total iron were highest in the LPS + Fer-1 group, followed by the LPS + Fer-1 group, and Fer-1/control group (Fig. 6b-d). Similar to the in vitro experiment, the mRNA level of *Hepcidin* in the LPS group was also decreased by Fer-1 treatment in vivo (Fig. 6e). Furthermore, the expression of both SLC7A11 and GPX4 was increased in the LPS + Fer-1 group compared with the LPS group (Fig. 6f). Collectively, these results indicated that Fer-1 alleviates LPS-induced ALI via inhibiting ferroptosis, which plays a key role in LPS-induced ALI.

## Discussion

Even though the LPS-induced ALI model has been established for years and widely used in pre-clinical studies, the accurate mechanisms of LPS-induced ALI are not yet fully understood [7, 36]. Researchers have found that the excessive accumulation of ROS and a burst of inflammatory cytokines (e.g. IL-6 and TGF-β) hold an important position in the pathogenesis of lung injury, and cell death is also considered as a key issue in LPS-induced ALI. Apoptosis has long been regarded as the major form of cell death [36–38]. However, because the accumulation of ROS exists in the LPS-induced ALI, it could be possible that there are still other types of cell death in ALI besides apoptosis. Ferroptosis is kind of iron-dependent programmed cell death, regulated by lipid oxidation. This cell death is implicated in many disease pathologies, such as neurodegeneration, inflammation, and ischemia-reperfusion injury [24, 39–41]. In this study, we mainly explored the position of ferroptosis in LPS-induced ALI. Our results indicated that the LPS could induce ferroptosis in lung cells in vitro and in vivo, and the ferroptosis inhibitor showed therapeutic action against LPS-induced ALI, providing a novel insight into the cell death pathways in LPS-induced ALI.

**Fig. 7:**
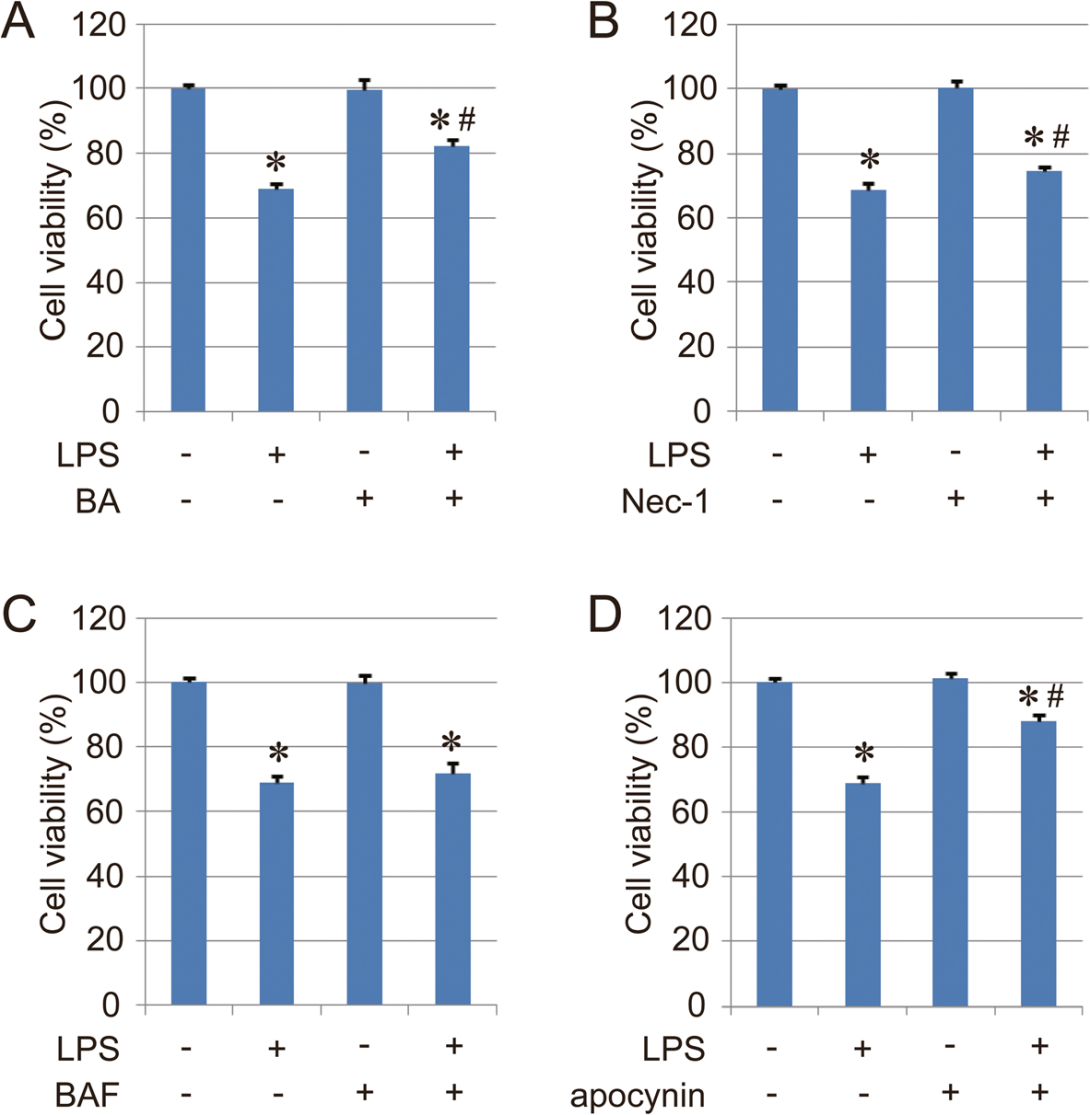
Cell viability of BEAS-2B cells treated with LPS and different inhibitors. Bongkrekic acid (BA, 20 μM, apoptosis inhibitor), necrostatin-1 (Nec-1, 50 μM, necroptosis inhibitor), bafilomycin A1 (BAF, 50 nM, autophagy inhibitor), and apocynin (200 μM, inflammation inhibitor) were used to rescue cell injury induced by LPS. Results are expressed as means±SEM (*n* = 3). *: *p* < 0.05 compared with the control group. #: *p* < 0.05 compared with the LPS group

Moreover, some researchers have demonstrated that all of apoptosis, necroptosis, autophagy, and inflammation were involved in LPS-induced ALI [2, 7, 42, 43]. To further evaluate each contribution to LPS-induced ALI, the LPS-induced BEAS-2B cell injury model was established in vitro, and the cells were treated with Fer-1 (2 μM, ferroptosis inhibitor), bongkrekic acid (BA, 20 μM, apoptosis inhibitor), necrostatin-1 (Nec-1, 50 μM, necroptosis inhibitor), bafilomycin A1 (BAF, 50 nM, autophagy inhibitor), and apocynin (200 μM, inflammation inhibitor) to rescue cell viability. The results indicated that all of the inhibitors showed a rescue effect except bafilomycin A1, and apocynin had the best effect in the LPS-induced injury model in vitro compared with other inhibitors (Fig. 2a and Fig. 7a-d). Our study mainly indicated that ferroptosis was also involved in LPS-induced ALI. It could be possible that the therapeutic mechanisms of these inhibitors are related to each other. For example, the treatment with Fer-1 could decrease the levels of BAL proinflammatory cytokines IL-6 and TNF-α (Fig. 5e-f). Therefore, it is very hard to evaluate the ratio of contribution from ferroptosis, inflammation, apoptosis and necroptotic cell death so far. Maybe more specific and effective models are still necessary for the analysis of each contribution to LPS-induced ALI in vivo.

Fer-1 is the first ferroptosis inhibitor, and is widely used in vitro and in vivo [44–47]. The function of Fer-1 against ferroptosis mainly depends on the inhibition of lipid peroxidation. Recently, another group indicated that the anti-ferroptotic effect of fer-1 is mainly dependent on the scavenging of initiating alkoxyl radicals and other rearrangement products [48]. We found that the expression level of *HEPCIDIN* in the LPS group also could be decreased by Fer-1 treatment in vitro and in vivo (Fig. 2e, 3e and 6e), which could be a reason for the effect of Fer-1 on total iron level. However, whether this effect of Fer-1 on hepcidin expression and total iron levels is direct or indirect remains unclear, and the deep mechanisms still need more investigation in different models. Moreover, some researchers have noted that the in vivo function of Fer-1 is weaker than the function in vitro, because of the plasma and metabolic instability [49, 50]. Therefore, the development of a more stable and potent ferroptosis specific inhibitor is still necessary for the in vitro study in the field of ferroptosis. Recently, some researchers found that liproxstatin-1 (another ferroptosis inhibitor) is more stable than Fer-1, and liproxstatin-1 also did not interfere with other types of cell death [26, 49, 50]. In our study, Fer-1 was applied in both in vitro and in vivo models, and showed an obvious effect against ferroptosis. It might be possible that the effect would be further improved if liproxstatin-1 was used in our research. Also, Fer-1 was administered after LPS challenge via tail vein injection herein. Therefore, Fer-1 in venous blood will enter the pulmonary circulation and work on lung tissue after injection immediately, which will enhance the therapeutic action of Fer-1 compared with intraperitoneal injection or oral administration. Even though other ferroptosis inhibitors may have a longer half-life in vivo, no comparative analysis has been performed in detail in a lung injury model so far. Our results mainly indicated that Fer-1 exerts therapeutic action against ALI, and it is also possible that the parameters at a shorter time point (less than 16 h after the injection of Fer-1) could show a better therapeutic effect. Of course, this hypothesis still needs our further exploration.

Numerous studies have demonstrated the crucial role of the infiltration of inflammatory cells, which is caused by the inflammatory cytokines during the progress of LPS-induced ALI. Furthermore, some researchers also noted that the increased infiltration of inflammatory cells could enhance the synthesis and accumulation of ROS in lung tissues [2, 4, 6, 12, 13]. In our study, the levels of IL-6 and TNF-α in BAL were increased in the LPS-induced ALI, and treatment with the ferroptosis inhibitor Fer-1 decreased the levels of both IL-6 and TNF-α in BAL, indicating the relationship between ferroptosis and inflammatory cytokines. Some studies have indicated that lipid peroxidation in ferroptosis can promote the inflammation and regulate the level of different inflammatory cytokines [39, 51, 52], which is consistent with our results. Moreover, the excessive accumulation of ROS also causes oxidative damage and an inflammatory response in lung tissues [53–55]. Ferroptosis is mainly induced by the failure of membrane lipid repair, and further leads to the increase of ROS on the membrane lipids. Therefore, the excessive accumulation of ROS caused by LPS treatment could be associated with the ferroptosis in LPS-induced ALI, and ROS-induced oxidative damage may also be regarded as a key causative factor in the different inflammatory events involved in ALI. However, the detailed role of ferroptosis and ROS in the inflammatory micro-environment still needs be explored intensively.

SLC7A11 and GPX4 are considered as the central regulators of ferroptosis, and reduced levels of GPX4 and SLC7A11 are always regarded as markers of ferroptosis [56–58]. In our study, we found that both SLC7A11 and GPX4 were clearly decreased in the LPS-induced ALI model, suggesting that ferroptosis occurred during the process of LPS-induced ALI. Moreover, the administration of Fer-1 inhibited LPS-induced ALI and increased the protein levels of both SLC7A11 and GPX4 in lung cells and tissues. These results further suggested that ferroptosis holds an important position during LPS-induced ALI, and a ferroptosis inhibitor should have an effective therapeutic action and reduce the histological alteration in ALI mice.

## Conclusions

In conclusion, our results indicated that ferroptosis played an important role in LPS-induced ALI, and Fer-1 alleviated LPS-induced ALI and the inflammatory response in vivo effectively via regulating ferroptosis. Therefore, our study demonstrated that a novel form of regulated cell death, ferroptosis, occurred in LPS-induced ALI, which was totally distinct from the classical cell apoptosis; that ferroptosis holds the potential to become a novel therapeutic target in ALI; and that a ferroptosis inhibitor might be an effective kind of drug for ALI patients.

## Supplementary information

**Additional file 1: Supplementary Fig. 1**. Uncropped blots for images shown throughout the paper.

**Additional file 2: Supplementary Fig. 2**. Raw images of HE staining in Fig. 4a.

## Data Availability

All data generated or analyzed during this study are included in this published article and its supplementary information files.

## Abbreviations

4-HNE: 4-hydroxynonenal
Abs: Absorbances
ALI: Acute lung injury
ATCC: American type culture collection
BA: Bongkrekic acid
BAF: Bafilomycin A1
BAL: Bronchoalveolar lavage
Fer-1: Ferrostatin-1
LPS: Lipopolysaccharide
MDA: Malondialdehyde
Nec-1: Necrostatin-1
Ptgs2: Prostaglandin endoperoxide synthase 2
qPCR: Real-time quantitative PCR
RIPA: Radioimmunoprecipitation assay lysis buffer
ROS: Reactive oxygen species
