# Supplementary figures and images for: Ferrostatin-1 alleviates lipopolysaccharide-induced acute lung injury via inhibiting ferroptosis
Source: Cell Mol Biol Lett. 2020 Feb 27;25:10. doi: 10.1186/s11658-020-00205-0 (PMC7045739; doi:10.1186/s11658-020-00205-0)

# Supplementary Figure 1

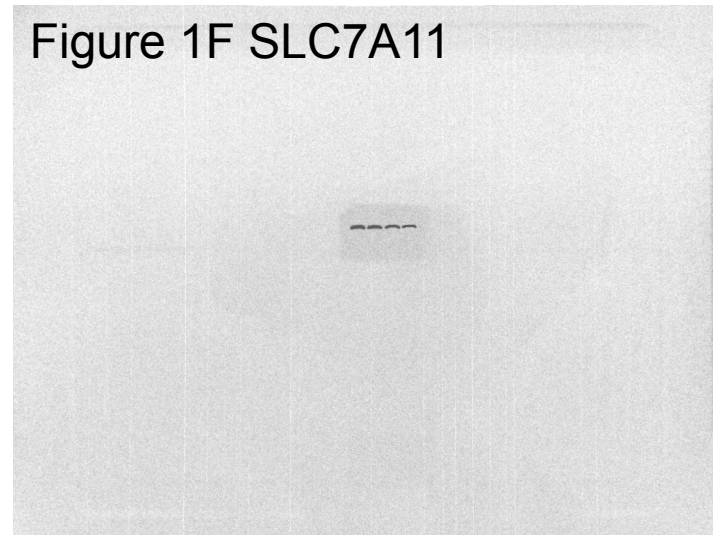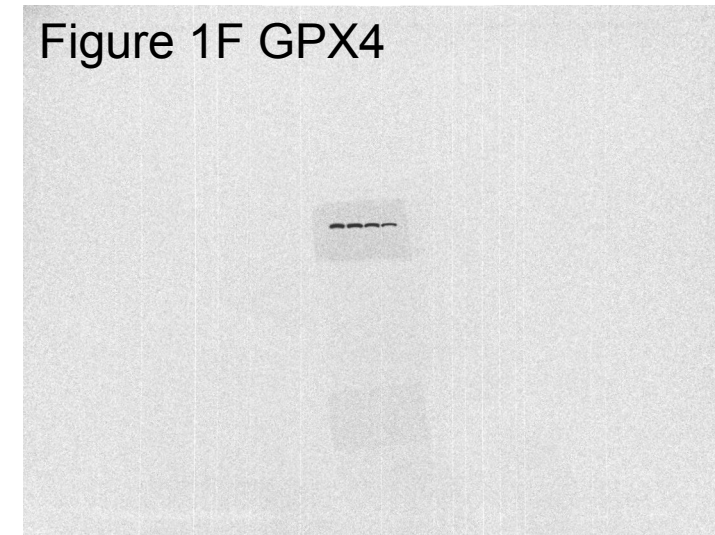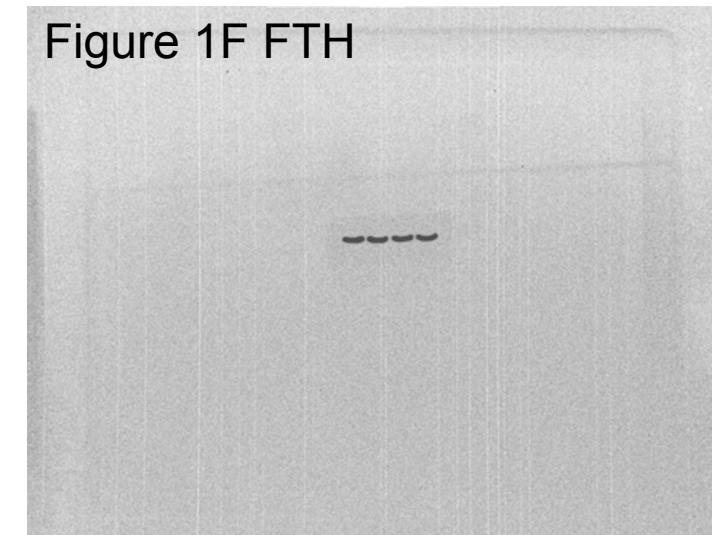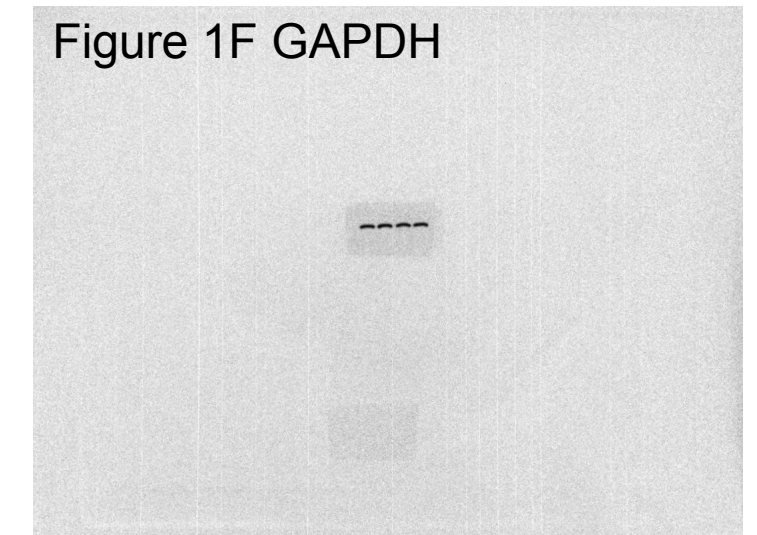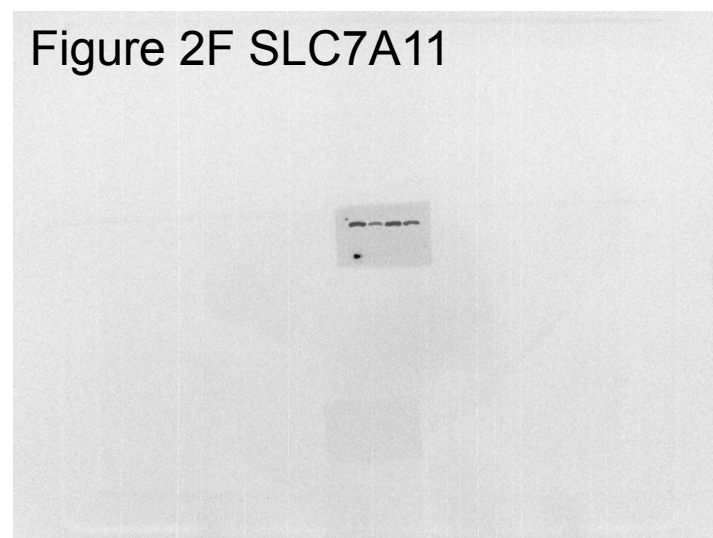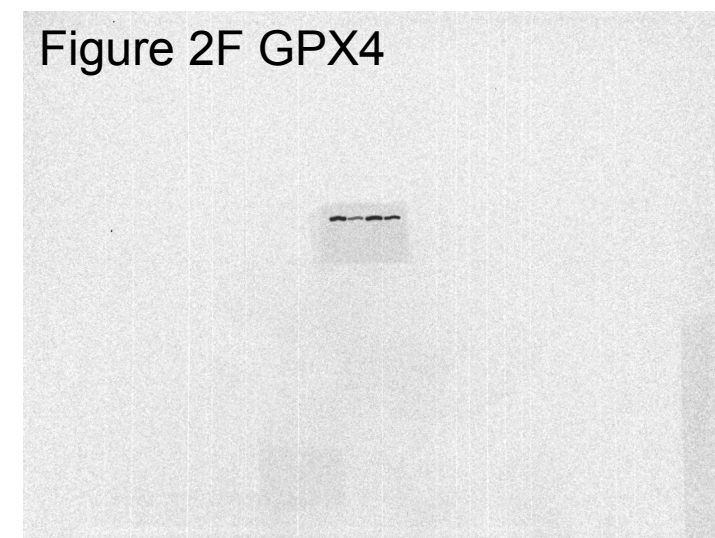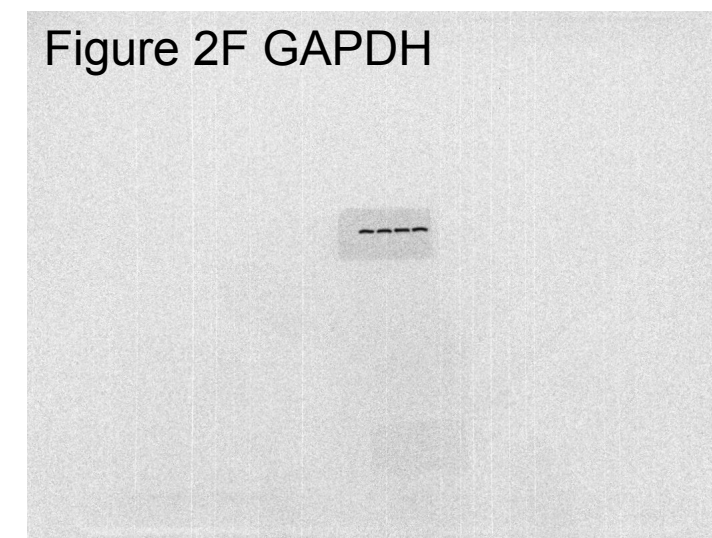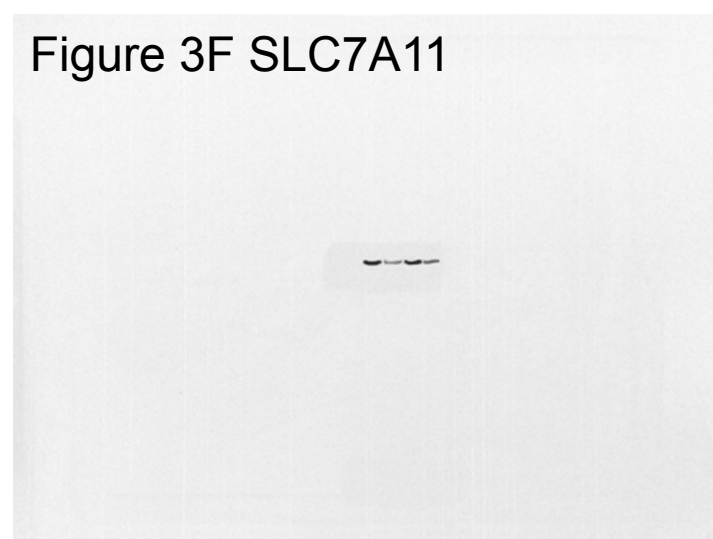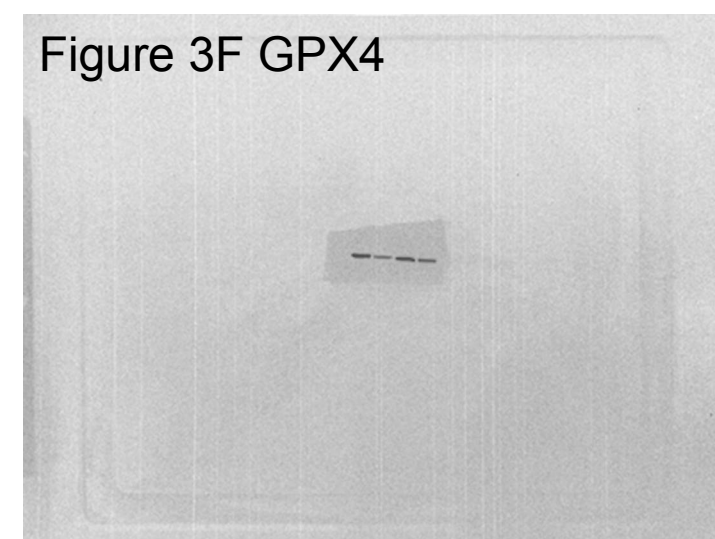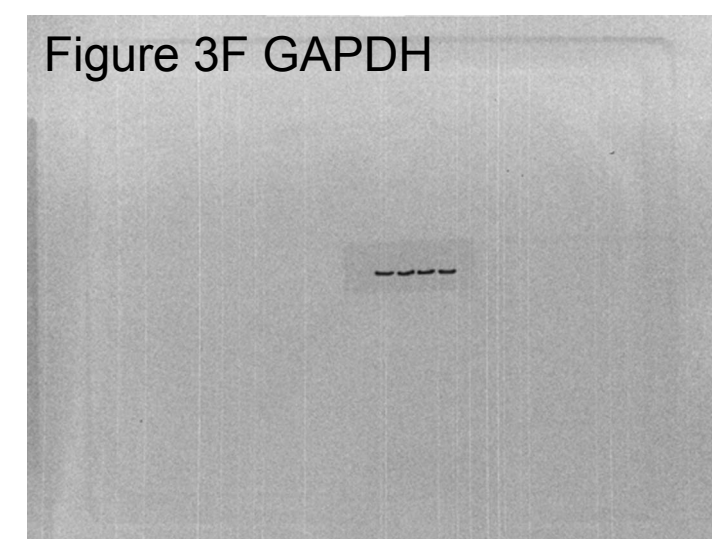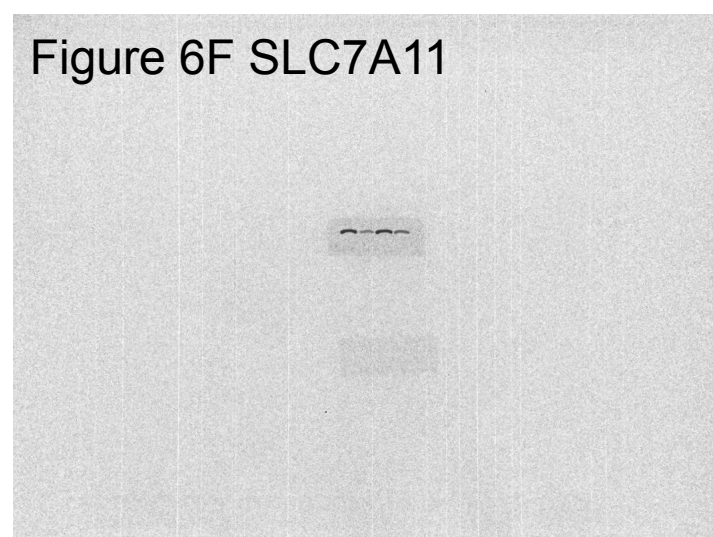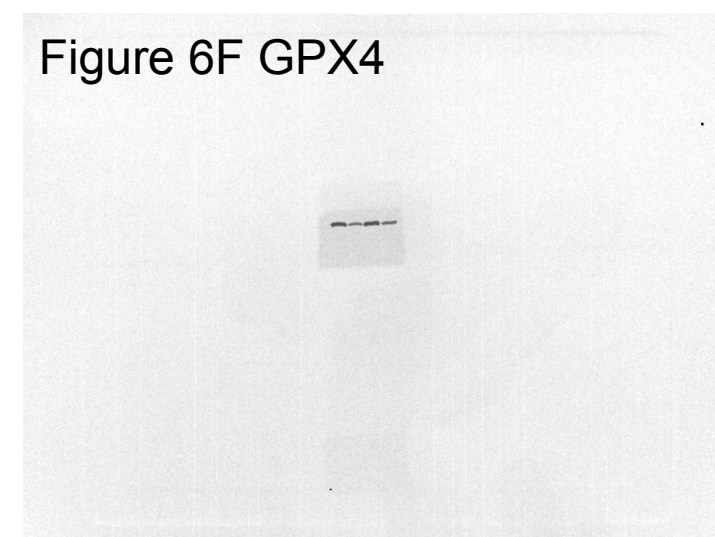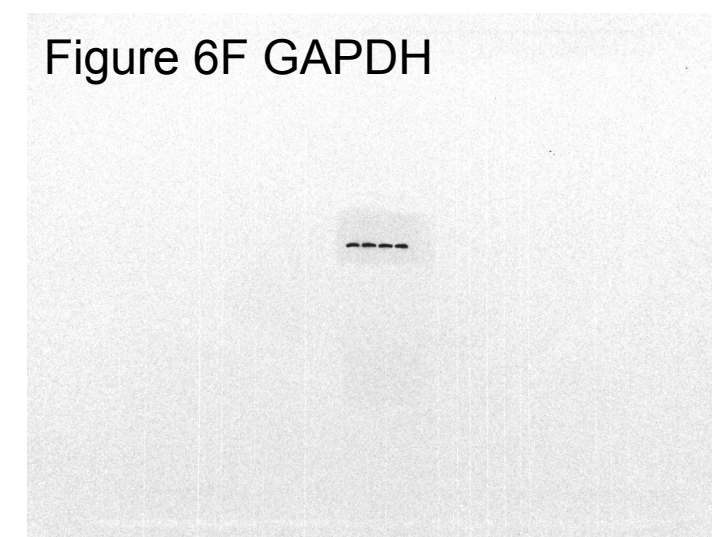

Supplement: Supplementary file 1 — Additional file 1: Supplementary Fig. 1. Uncropped blots for images shown throughout the paper. [file 11658_2020_205_MOESM1_ESM.pdf]

# Supplementary Figure 2

Ctrl

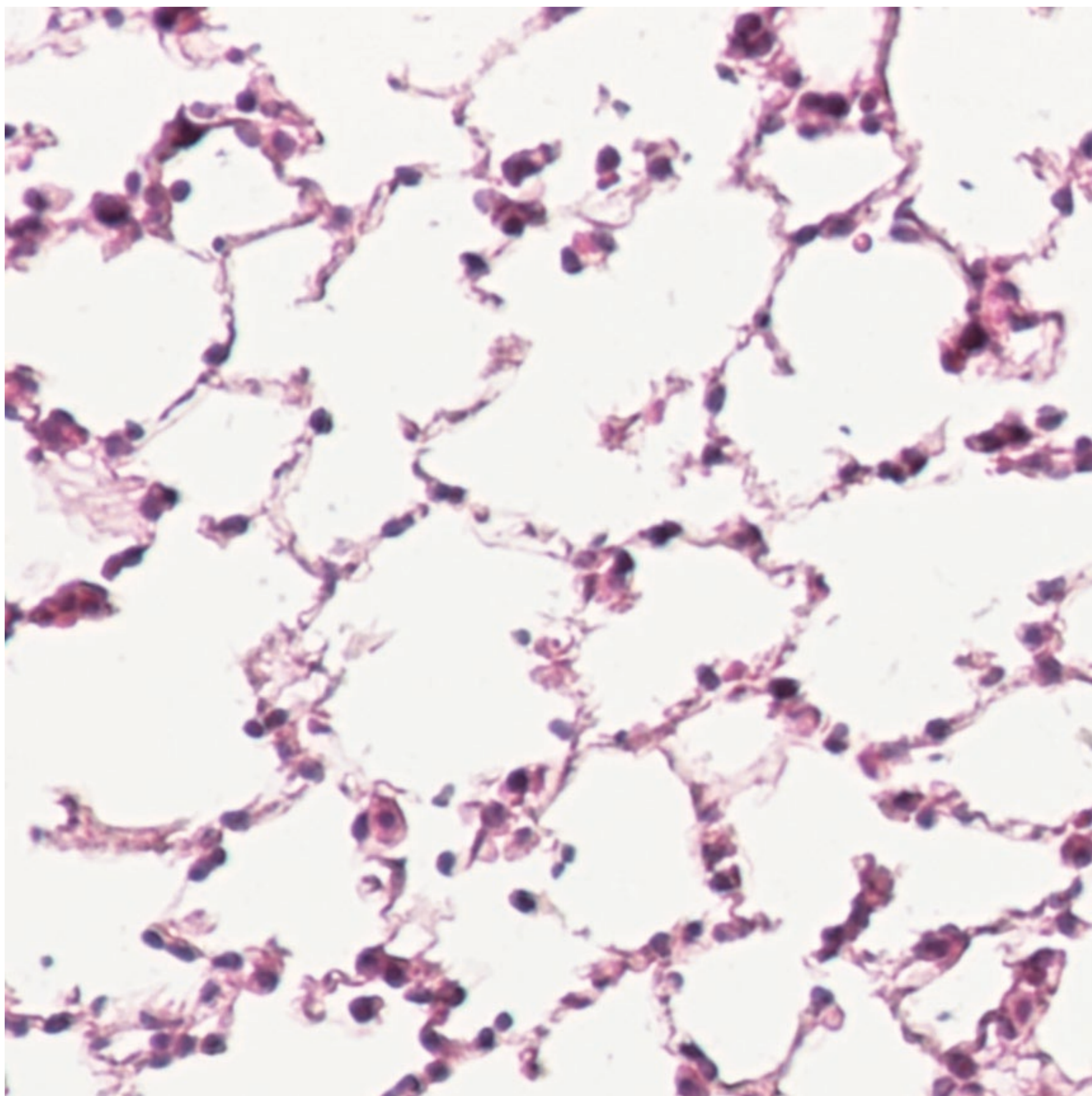

LPS

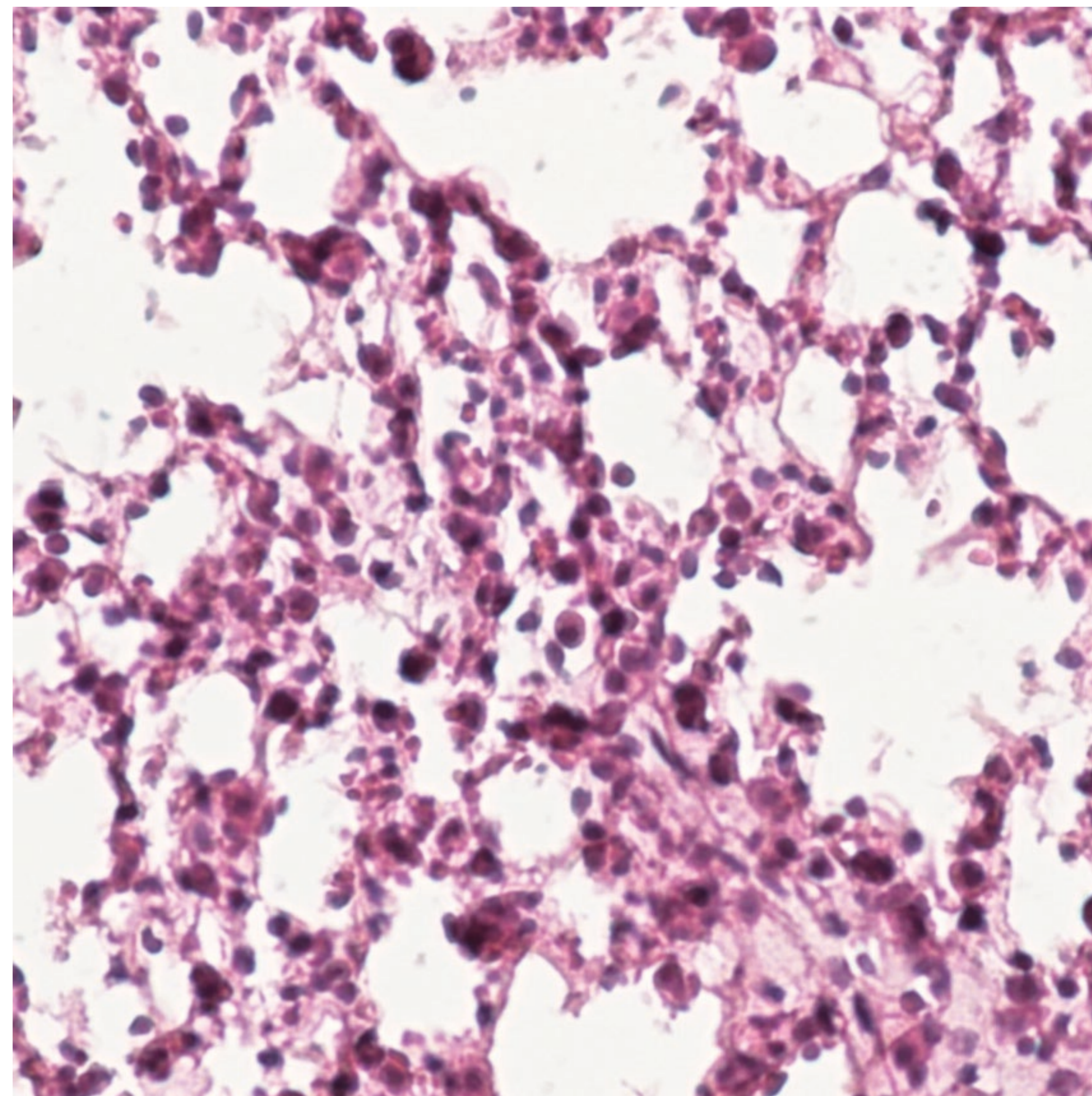

Fer-1

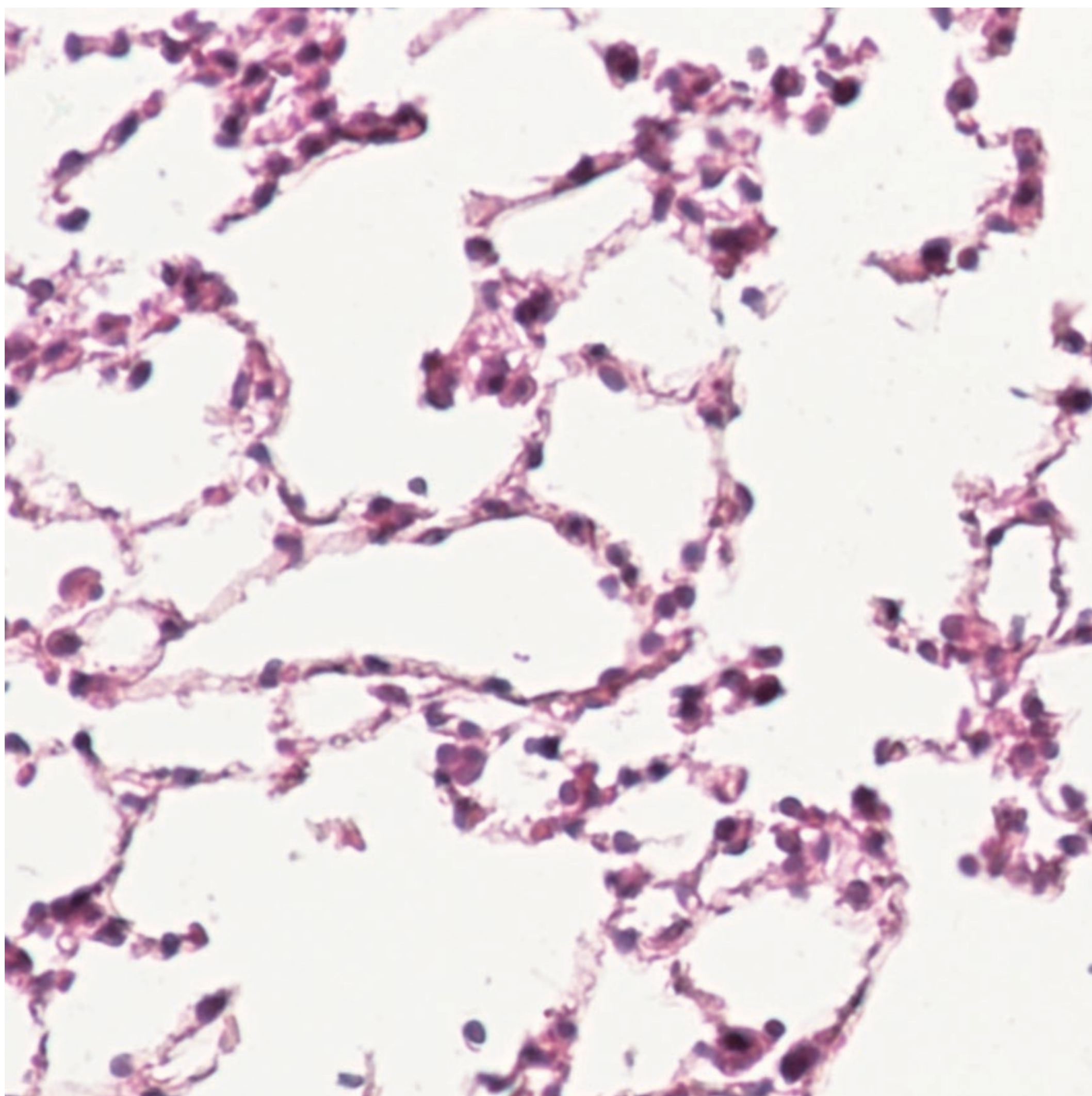

LPS+Fer-1

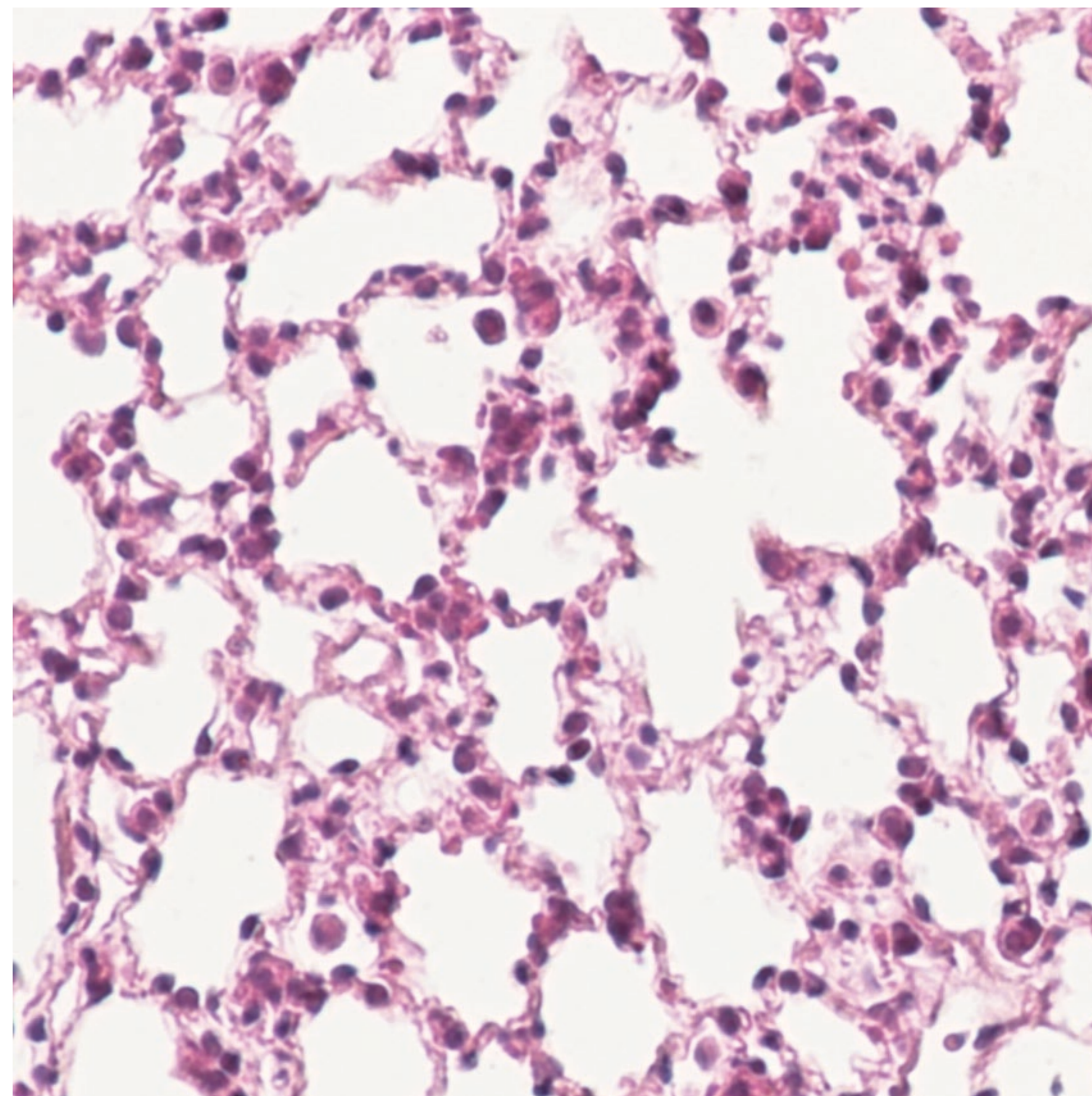

Supplement: Supplementary file 2 — Additional file 2: Supplementary Fig. 2. Raw images of HE staining in Fig. 4a. [file 11658_2020_205_MOESM2_ESM.pdf]
